# Supplementary material for: Characterizing cell-type spatial relationships across length scales in spatially resolved omics data
Source: Nat Commun. 2025 Jan 3;16:350. doi: 10.1038/s41467-024-55700-1 (PMC11699133; doi:10.1038/s41467-024-55700-1)
Supplement: Supplementary file 3 — Supplementary Software 1 [file 41467_2024_55700_MOESM3_ESM.zip › CRAWDAD/plots/spleen/3_spleen_pkhl_tsne_labs.pdf]

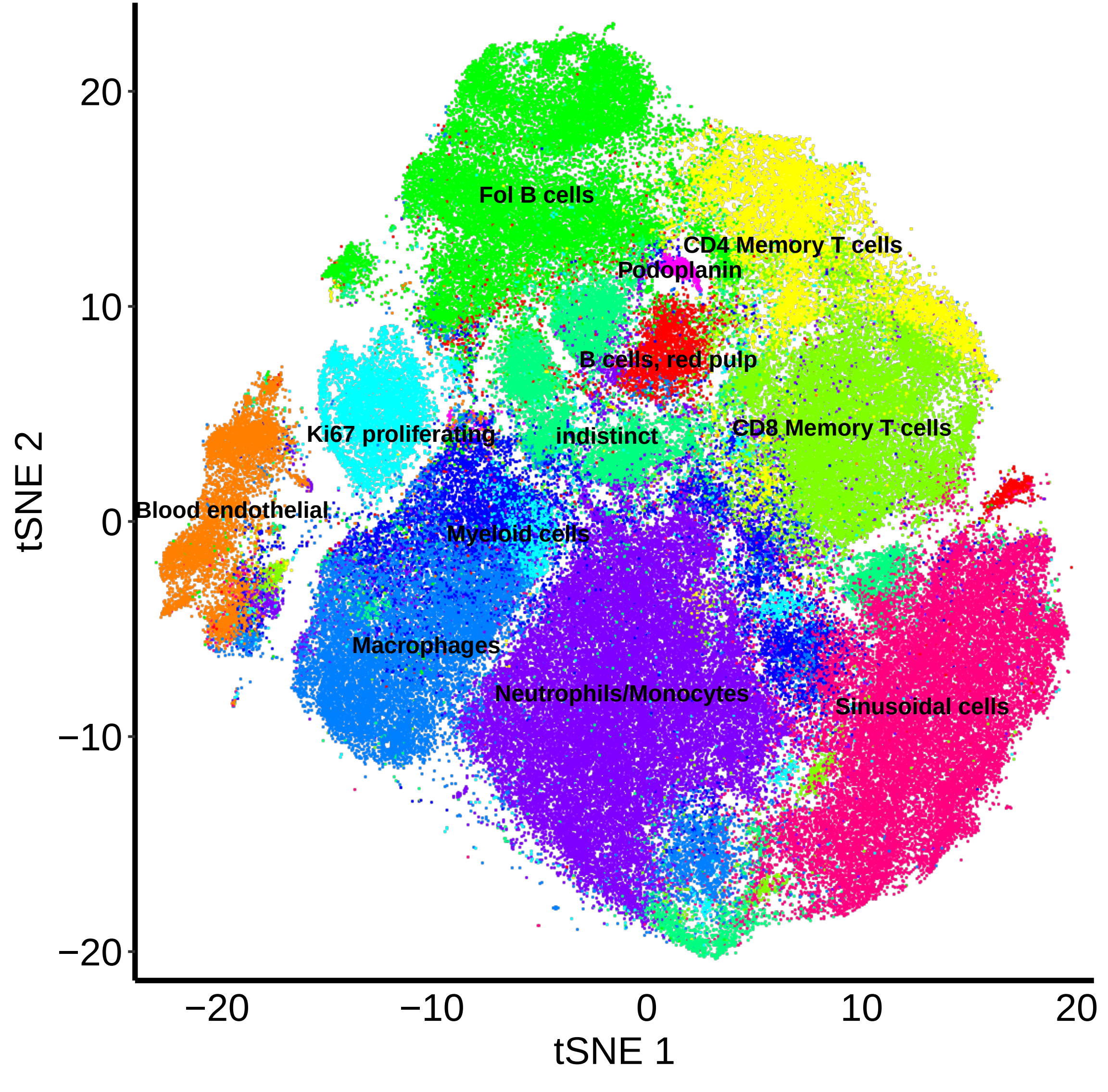
Fol B cells
CD4 Memory T cells
Podoplanin
B cells, red pulp
Ki67 proliferating
indistinct
CD8 Memory T cells
Blood endothelial
Myeloid cells
Macrophages
Neutrophils/Monocytes
Sinusoidal cells
20
10
0
−10
−20
−20
−10
0
10
20
tSNE 2
tSNE 1
